# Health Literacy and Preventive Behaviors Modify the Association between Pre-Existing Health Conditions and Suspected COVID-19 Symptoms: A Multi-Institutional Survey

**DOI:** 10.3390/ijerph17228598

**Published:** 2020-11-19

**Authors:** Tan T. Nguyen, Nga T. Le, Minh H. Nguyen, Linh V. Pham, Binh N. Do, Hoang C. Nguyen, Huu C. Nguyen, Tung H. Ha, Hung K. Dao, Phuoc B. Nguyen, Manh V. Trinh, Thinh V. Do, Hung Q. Nguyen, Thao T. P. Nguyen, Nhan P. T. Nguyen, Cuong Q. Tran, Khanh V. Tran, Trang T. Duong, Thu T. M. Pham, Tuyen Van Duong

**Affiliations:** 1 Department of Orthopedics, Can Tho University of Medicine and Pharmacy, Can Tho 941-17, Vietnam; nttan@ctump.edu.vn; 2 Director Office, Can Tho University of Medicine and Pharmacy Hospital, Can Tho 941-17, Vietnam; 3 Allied Health & Human Performance, Higher Degree Research, University of South Australia, Adelaide, SA 5000, Australia; lengahp0201@gmail.com; 4 International Ph.D. Program in Medicine, Taipei Medical University, Taipei 110-31, Taiwan; d142108015@tmu.edu.tw; 5 Department of Pulmonary & Cardiovascular Diseases, Hai Phong University of Medicine and Pharmacy Hospital, Hai Phong 042-12, Vietnam; pvlinh@hpmu.edu.vn; 6 Director Office, Hai Phong University of Medicine and Pharmacy Hospital, Hai Phong 042-12, Vietnam; 7 Department of Infectious Diseases, Vietnam Military Medical University, Hanoi 121-08, Vietnam; nhubinh.do@vmmu.edu.vn; 8 Division of Military Science, Military Hospital 103, Hanoi 121-08, Vietnam; 9 Director Office, Thai Nguyen National Hospital, Thai Nguyen City 241-24, Vietnam; nguyenconghoang@tnmc.edu.vn; 10 President Office, Thai Nguyen University of Medicine and Pharmacy, Thai Nguyen City 241-17, Vietnam; 11 Department of Thoracic and Cardiovascular Surgery, E Hospital, Hanoi 113-08, Vietnam; bacsyhuu@gmail.com; 12 Director Office, E Hospital, Hanoi 113-08, Vietnam; 13 Director Office, General Hospital of Agricultural, Hanoi 125-16, Vietnam; hahuutung.200564@gmail.com; 14 Director Office, Bac Ninh Obstetrics and Pediatrics Hospital, Bac Ninh 161-23, Vietnam; daokhachung2000@yahoo.com; 15 Director Office, Kien An Hospital, Hai Phong 046-09, Vietnam; nguyenbatuankiet@gmail.com; 16 Director Office, Quang Ninh General Hospital, Quang Ninh 011-08, Vietnam; trinhmanhqnsyt@gmail.com; 17 Director Office, Bai Chay Hospital, Quang Ninh 011-21, Vietnam; dovanthinhhscc@gmail.com; 18 Director Office, Quang Ninh Obstetrics and Pediatrics Hospital, Quang Ninh 011-24, Vietnam; bshungbvqn@gmail.com; 19 Health Management Training Institute, Hue University of Medicine and Pharmacy, Thua Thien Hue 491-20, Vietnam; ntpthao.hmti@huemed-univ.edu.vn; 20 Department of Health Economics, Corvinus University of Budapest, 1093 Budapest, Hungary; 21 General Planning Department, Da Nang Oncology Hospital, Da Nang 506-06, Vietnam; bsnhanbvub@gmail.com; 22 Director Office, Thu Duc District Health Center, Ho Chi Minh City 713-10, Vietnam; quoccuong.mph@gmail.com; 23 Faculty of Health, Mekong University, Vinh Long 852-16, Vietnam; 24 Director Office, Hospital District 2, Ho Chi Minh City 711-13, Vietnam; tvkhanh.q2@tphcm.gov.vn; 25 Nursing Office, Tan Phu District Hospital, Ho Chi Minh City 720-16, Vietnam; duongtrang7273@gmail.com; 26 Faculty of Public Health, Hai Phong University of Medicine and Pharmacy, Hai Phong 042-12, Vietnam; phamminhthu.ytcc@gmail.com; 27 School of Public Health, College of Public Health, Taipei Medical University, Taipei 110-31, Taiwan; 28 School of Nutrition and Health Sciences, Taipei Medical University, Taipei 110-31, Taiwan

**Keywords:** suspected COVID-19 symptoms, coronavirus, health literacy, preventive behaviors, mask wearing, hand washing, physical distancing, pre-existing health conditions

## Abstract

People with pre-existing health conditions (PEHC) are vulnerable to viral infection while health literacy (HL) and preventive behaviors (PB) have been shown to benefit people during the COVID-19 pandemic. The aim of this study was to examine the association between PEHC and suspected COVID-19 symptoms (S-COVID-19-S), and to investigate the modification effect of HL and PB. A cross-sectional study was conducted on 8291 participants visiting outpatient departments at 18 hospitals and health centers across Vietnam from 14 February to 31 May 2020. Data were collected regarding participant’s characteristics, HL, PB, PEHC, and S-COVID-19-S. Regression models were used for analyzing the associations. Results showed that people with PEHC had a 3.38 times higher likelihood of having S-COVID-19-S (odds ratio, OR, 3.38; 95% confidence interval, 95% CI, 3.01, 3.79; *p* < 0.001). In comparison to participants without PEHC and with the lowest HL score, those with PEHC and one HL score increment had a 7% lower likelihood of having S-COVID-19-S (OR, 0.93; 95% CI, 0.92, 0.94; *p* < 0.001). In comparison to participants without PEHC and not adhering to mask wearing, those with PEHC and adhering to mask wearing had a 77% lower likelihood of having S-COVID-19-S (OR, 0.23; 95% CI, 0.16, 0.32; *p* < 0.001). Higher HL and adherence to mask wearing can protect people from having S-COVID-19-S, especially in those with PEHC.

## 1. Introduction

The coronavirus disease 2019 (COVID-19) pandemic causes devastating healthcare and economic impacts on countries [1,2,3,4]. Pre-existing health conditions (PEHC) such as diabetes and cardiovascular disease (CVD) were pointed out to increase the risk of COVID-19 infection [5,6,7], and worsen the prognosis of COVID-19 infection [8,9]. People with COVID-19 and PEHC are more susceptible to die from COVID-19 [10,11].

Coronavirus has been indicated to be transmitted via the contact with virus-containing secretions expelled through expiratory human activities of infected persons such as breathing, talking, sneezing, coughing, and singing [12,13]. Thus, it is a matter of great concern and necessary to explore preventive factors that help to reduce the occurrence of viral infection.

Health literacy (HL) is defined as individuals’ perceptions, knowledge, and ability to understand, access, appraise, and apply health information into disease prevention and treatment in their life course in order to improve their quality of life [14]. Low HL is associated with worse health decisions and outcomes [15,16,17]. People with low HL have difficulties communicating effectively with health care providers [18]. This requires more efforts from physicians to communicate in colloquial language to promote patients’ involvement in shared decision-making [19]. People with high HL have been shown to be more likely to adopt protective methods and have the preparedness to get protected from the COVID-19 infection [15].

Preventive behaviors such as mask wearing, hand washing, and physical distancing have been recommended by World Health Organization (WHO) [12] and Centers for Disease Control and Prevention (CDC) [20] as effective personal methods to protect people from COVID-19 infection. These preventive behaviors have been indicated to significantly reduce the risk of viral transmission and infection in previous studies [21,22,23]. A systematic review and meta-analysis of 172 observational studies across 16 countries and six continents supports the physical distancing and face masking that prove to be beneficial for preventing the infection [24]. In addition, wearing mask and washing hands with soap helps reduce the transmission of viruses [25].

Health literacy and preventive behaviors have shown the protective effects in containing the COVID-19 pandemic. However, their roles on controlling the infection in people with pre-existing health conditions remain to be explored. Therefore, in this study, we examined the association between pre-existing health conditions (PEHC) and suspected COVID-19 symptoms (S-COVID-19-S), and we investigated the effect modification of health literacy (HL) and preventive behaviors (PB) on the association between PEHC and S-COVID-19-S among outpatient department visitors in 18 hospitals and health centers across Vietnam during the pandemic.

## 2. Methods

### 2.1. Study Design

A cross-sectional study was conducted on adults visiting the outpatient department (OPD) at 18 hospitals and health centers across Vietnam from 14 February to 31 May 2020. The study was reviewed and approved by the Institutional Ethical Review Committee of Hanoi University of Public Health, Vietnam (IRB No. 029/2020/YTCC-HD3 for the first period from 14 February to 31 March 2020, and IRB No. 133/2020/YTCC-HD3 for the second period from 1 April to 31 May 2020).

### 2.2. Study Sample

Adults who visited the OPD, aged from 18 to 85 years old with Vietnamese listening and reading proficiency, were invited to join our survey. We excluded individuals with any emergency condition (e.g., positive with COVID-19, injuries from traffic accident, appendicitis, etc.), having a psychotic disorder, dementia, or blindness. The data collection procedures were described in a previous publication [26].

A total of 8291 participants from 18 hospitals and health centers across Vietnam, including 1028 from Military Hospital 103, 183 from E hospital, and 300 from General Hospital of Agricultural, in Hanoi; 469 from Thai Nguyen National Hospital, in Thai Nguyen Province; 500 from Bac Ninh Obstetrics and Pediatrics Hospital, in Bac Ninh city; 982 from Hai Phong University of Medicine and Pharmacy Hospital, 492 from Kien An Hospital, and 484 from Kien Thuy District Health Center, in Haiphong city; 309 from Quang Ninh General Hospital, 364 from Bai Chay Hospital, and 280 from Quang Ninh Obstetrics and Pediatrics Hospital, in Quang Ninh province; 495 from Trieu Phong District Health Center, Quang Tri province; 421 from Da Nang Oncology Hospital, in Da Nang city; 489 from Thu Duc District Hospital, 497 from Thu Duc District Health Center, 248 from Hospital District 2, and 242 from Tan Phu District Hospital, in Ho Chi Minh city; and 508 from Can Tho University of Medicine and Pharmacy Hospital, in Can Tho city.

### 2.3. Instruments and Measurements

#### 2.3.1. Participant Characteristics

Participants were asked for socio-demographic information, including age (18–39, 40–64, 65–80 years old), gender (women, men), education attainment (junior high school or below, senior high school, college/university level or above), occupation (employed, own business, others), ability to pay for medication (very or fairly difficult, very or fairly easy to pay), and social status (low, middle and high). Body height (cm) and weight (kg) of participants was measured. Body mass index was calculated as (weight (kg))/(height (m))^2^.

#### 2.3.2. Health Literacy

The short version of the health literacy questionnaire (HLS-SF12) consisting of 12 items was used to assess the health literacy of participants in our study. This health literacy questionnaire has been validated and used for the general population (aged ≥ 15 years, both sexes) in Asia [27], including Vietnam [28,29,30,31,32,33,34]. Participants responded to each item on 4-point Likert scales from 1 = very difficult to 4 = very easy. The HL index was standardized to a unified metric from 0 to 50, with a higher score that represents better health literacy, using the following Formula (1):

*Index* = (*Mean* − 1) × (50/3) (1)

where *Index* is the calculated HL index, *Mean* is the mean of all participating items for each individual, 1 is the possible minimum value of the mean (leading to a minimum value of the index equals 0), 3 is the range of the mean, and 50 is the chosen maximum value of the new metric. A higher HL index indicates a better HL level [27,35].

#### 2.3.3. Preventive Behaviors

Participants were asked about their performance of preventive behaviors, including washing hands with soap or sanitizer when touching objects suspected to be contaminated or after going outside [20,36], wearing mask when going outside [20,36], and performing physical distancing (at least 2 m or 6 feet to people around) [37] since the COVID-19 outbreak started from the 23 January 2020 in Vietnam. The frequency of performing each behavior was rated on five levels (never, rarely, occasionally, often, and always/usually). Always/usually performing each preventive behavior during the COVID-19 outbreak was defined as adherence to that behavior [20]. Not always/usually (never, rarely, occasionally, or often) performing a preventive behavior was considered as non-adherence to that behavior.

#### 2.3.4. Pre-Existing Health Condition

Participants self-reported their pre-existing health condition (PEHC). The PEHC were assessed using the Charlson Comorbidity Index items [38], including myocardial infarction, congestive heart failure, peripheral disease, cerebrovascular disease, chronic pulmonary disease, diabetes without complications, depression, taking anticoagulants (for cardiovascular disease, thrombosis), hemiplegia, diabetes with complications, moderate or severe renal disease, tumor without metastasis, moderate or severe liver disease, solid metastatic tumor, and HIV/AIDS. Participants with one or more of these conditions when presented at the OPD were classified as having PEHC.

#### 2.3.5. Suspected COVID-19 Symptoms

Participants reported their health problems that are similar symptoms of COVID-19, including fever, cough, dyspnea, myalgia, fatigue, sputum production, confusion, headache, sore throat, rhinorrhea, chest pain, hemoptysis, diarrhea, and nausea/vomiting [39]. Individuals having one of those symptoms when visiting the OPD were classified as having suspected COVID-19 symptoms or symptoms like COVID-19 [33]. This did not imply that participants were suspected of having COVID-19.

### 2.4. Statistical Analysis

Firstly, the Chi-square test was used to compare the proportion of S-COVID-19-S by different categorical variables, including participant characteristics (age, gender, marital status, education, occupation, social status, ability to pay medication, BMI), PEHC, and PB (mask wearing, hand washing, physical distancing) in studied participants. The analysis of variance (ANOVA) test was used to examine the distribution of continuous variables, including the HL index. Secondly, unadjusted and adjusted logistic regression models were performed to explore the associations of PEHC, PB, and HL with S-COVID-19-S. The factors significantly associated with having S-COVID-19-S at *p* < 0.05 via bivariate logistic regression were considered as confounding factors and were adjusted in the multivariate model. To avoid multicollinearity, the correlations between covariates were examined. If the Spearman’s rho ≥ 0.3 exists between covariates, one representative variable was selected into the multivariate model. Finally, the interactions between HL, PB, and PEHC on having S-COVID-19-S were examined using bivariate and multivariate logistic regression models. All statistical analysis was performed by SPSS Version 20.0 (IBM Corp, Armonk, NY, USA), and a *p* < 0.05 was set as significance.

## 3. Results

### 3.1. Characteristics of Studied Participants

Out of all participants, the proportions of PEHC, adherence to mask wearing, hand washing, and social distancing were 22.6%, 44.2%, 24.3%, and 20.6%, respectively. The proportion of S-COVID-19-S was 37.7% (3129/8291). This proportion was significantly differed by participants’ age, marital status, education, occupation, ability to pay, social status, and preventive behaviors. People with S-COVID-19-S had a lower health literacy score than those without S-COVID-19-S (Table 1).

### 3.2. Associations between Health Literacy, Preventive Behaviors, and Suspected COVID-19 Symptoms

In the bivariate logistic analysis, participants with PEHC had a higher likelihood of having S-COVID-19 while those who adhered to PB had lower likelihood of having S-COVID-19-S than their counterparts (*p* < 0.001; Table 2). The association between associated factors and S-COVID-19-S was fully presented in Table S1. Age, education, marital status, occupation, ability to pay for medication, social status, and BMI were found to be significantly associated with the risk of having S-COVID-19-S (*p* < 0.05). To avoid multicollinearity, Spearman correlations among factors were examined. A moderate correlation between age and education level (Spearman’s rho: −0.41), and between age and marital status (Spearman’s rho: 0.38) were found (Table S2). Hence, education attainment and marital status were excluded, and finally, age, occupation, ability to pay for medication, social status, and BMI were selected into multivariate model.

After adjusting for selected variables, PEHC was significantly associated with a 3.38 times higher likelihood of S-COVID-19-S (odds ratio, OR, 3.38; 95% confidence interval, 95% CI, 3.01, 3.79; *p* < 0.001). Participants who adhered to mask wearing (OR, 0.36; 95% CI, 0.31, 0.41; *p* < 0.001), hand washing (OR, 0.38; 95% CI, 0.32, 0.45; *p* < 0.001), physical distancing (OR, 0.35; 95% CI, 0.29, 0.42; *p* < 0.001), and with 1-score increment of HL (OR, 0.95; 95% CI, 0.94, 0.96; *p* < 0.001) had a lower likelihood of having S-COVID-19-S, respectively (Table 2).

### 3.3. Effect Modifications of Health Literacy and Preventive Behaviors

In comparison to participants without PEHC and the lowest HL score, those with PEHC and lowest HL score had a 16.53 times higher likelihood of having S-COVID-19-S, while a 1-score increment of HL resulted in a 7% lower likelihood of having S-COVID-19-S (OR, 0.93; 95% CI, 0.92, 0.94; *p* < 0.001). Similarly, in comparison to participants without PEHC and not adhering to mask wearing, those with PEHC and not adhering to mask wearing had a 17.42 times higher likelihood of having S-COVID-19-S, while those with PEHC and adhering to mask wearing resulted in a 77% lower likelihood of having S-COVID-19-S (OR, 0.23; 95% CI, 0.16, 0.32; *p* < 0.001; Table 3). The modifying effect of adherence to hand washing or social distancing to lower the likelihood of having S-COVID-19-S was not found (*p* > 0.05; Table 3).

## 4. Discussion

In this study, we found that participants with pre-existing health conditions (PEHC) had a significantly higher risk of having S-COVID-19-S than ones without PEHC. Participants, who adhered to performing preventive behaviors and who had higher health literacy scores had a lower risk of having S-COVID-19-S compared to their counterparts. Even though we did not study the symptoms of COVID-19, the symptoms like COVID-19 could possibly help to explore the associations and raise the awareness of the public. A meta-analysis of 1558 patients with COVID-19 showed that patients with certain underlying medical conditions such as hypertension, diabetes, chronic obstructive pulmonary disease (COPD), cardiovascular disease (CVD), and cerebrovascular disease had a higher risk of having COVID-19 than those without these illnesses [6]. Besides, patients with comorbidities, such as diabetes mellitus or chronic obstructive pulmonary disease (COPD), were prone to develop more severe progression of COVID-19 infection [6,9].

Preventive behaviors such as wearing mask, washing hands with soap, and performing physical distancing were proven to be effective in reducing the COVID-19 transmission and infection. A systematic review collecting 172 observational studies across 16 countries and six continents proved that lengthened physical distancing of at least two meters in public places has more effectively reduced the transmission of SARS-CoV-2 and beta coronaviruses (SARS-CoV or MERS-CoV) compared to a closer distance that is less than two meters [24]. Wearing mask and washing hands limit the contact with virus-containing secretion from infected people, thus wearing mask and washing hands with soap have been strongly recommended in the COVID-19 pandemic to reduce the spread of SARS-CoV-2 [40]. Mask wearing and hand washing have also been shown to be preventive measures to reduce the transmission of viruses during the severe acute respiratory syndrome (SARS) [25,41] and novel influenza A (H1N1) pandemic [42].

Regarding health literacy (HL), a study of behavioral reactions to the H1N1 influenza pandemic showed that individuals who had perceptions of the effectiveness of preventive behaviors, such as washing hands with warm water and soap, using a napkin when sneezing, and avoidance responses such as avoiding crowd places and public transportation were more likely have a reduced risk of having the viral infection [43]. The possible explanation was that individuals with a high HL level possibly had more adequate and appropriate information and health knowledge [44,45,46,47], which assisted them to make better health decisions and adopt preventive methods to protect themselves from the disease during the pandemic [48]. Also, HL was indicated to be positively associated with better attitudes and preparedness for the outbreak during the early onset of COVID-19 [15]. In addition, people with higher health literacy had better health-related behaviors that may be a benefit to their health [29].

Importantly, we found that health literacy and preventive behaviors (mask wearing, hand washing, and physical distancing) have modification effects on reducing the risk of having S-COVID-19-S in patients with PEHC. The finding is similar with a previous study that higher health literacy was found to be associated with better adherence to infection prevention and control behaviors, and lower risk of having S-COVID-19-S in health care workers [30]. Health literacy was previously suggested as the driving factor between socioeconomic status and health outcomes [49] and between education level and health status [44]. Noticeably, mask wearing was found to be the most effective modifier to lower the risk of having S-COVID-19-S in patients with underlying medical condition (up to 77% reduction). Even though hand washing and physical distancing were shown to insignificantly modify the risk of having S-COVID-19-S in highly vulnerable people after being adjusted for socio-demographic factors, these preventive behaviors were still shown to have a potentially protective effect to protect people with PEHC from having suspected COVID-19 symptoms.

Our study is the first study to investigate modification effects of preventive factors (such as health literacy, preventive behaviors) on the risk of having S-COVID-19-S in patients with PEHC. However, in our cross-sectional study, it is not possible to explore the causal relationship between underlying medical condition, health literacy, and preventive behaviors and the risk of having S-COVID-19-S in the study. Besides, suspected COVID-19 symptoms and underlying medical condition of participants were self-reported. Although self-reporting is a common approach for collecting data in epidemiological and medical studies, this method may lead to recall bias due to recall error of the past events of participants [50]. Therefore, the findings should be interpreted with caution. Fortunately, a large sample size allows us to examine the associations and interactions that help to raise the phenomenon for the future research. Another limitation was that we cannot identify patients with symptoms of COVID-19 or symptoms like COVID-19 by asking them. Fortunately, there were no new confirmed cases in the studied hospitals and health centers during the data collection period [51].

Despite of remaining limitations, our study helps to explore the modifying effects of preventive factors (health literacy and preventive behaviors) mitigating the negative association between having an underlying medical condition and the risk of having S-COVID-19-S. In the limited scope of one study, we assessed the modifying effect of each preventive behavior separately, but not the combined effects of these behaviors. This approach, however, helps us to figure out the differences of the protective effect of each behavior in people with pre-existing health conditions, and helps to find out the most significantly effective modifier. In further studies, exploring the factors associated with the adherence to preventive behaviors, and the combined effects of preventive behaviors on having suspected COVID-19 symptoms is essential to help researchers and practitioners to have oversight of people’s awareness and knowledge during the viral pandemic.

## 5. Conclusions

People with pre-existing health conditions had a higher likelihood of having S-COVID-19-S. Fortunately, higher health literacy and better adherence to mask wearing were associated with lower likelihood of having suspected COVID-19 symptoms, especially in those with pre-existing health conditions. Strategic public health approaches are needed to promote health literacy and infection preventive behaviors (e.g., mask wearing, hand washing, and social distancing) that may help to contain the pandemic.

## Figures and Tables

**Table 1:** Characteristics of studied participants (*N* = 8291).

Variables	Total (*N* = 8291)	Without S-COVID-19-S ^†^ (*N* = 5162)	With S-COVID-19-S ^†^ (*N* = 3129)	*p* *
	*N* (%)	*N* (%)	*N* (%)	
Age, year				<0.001
18–39	3955 (47.7)	2830 (54.8)	1125 (36)	
40–59	2473 (29.8)	1487 (28.8)	986 (31.5)	
60–85	1863 (22.5)	845 (16.4)	1018 (32.5)	
Gender				0.837
Women	4890 (59)	3049 (59.1)	1841 (58.8)	
Men	3401 (41)	2113 (40.9)	1288 (41.2)	
Marital status				<0.001
Never married	1635 (19.8)	1223 (23.8)	412 (13.2)	
Ever married	6628 (80.2)	3924 (76.2)	2704 (86.8)	
Education attainment				0.017
Junior high school or below	2223 (26.9)	1361 (26.4)	862 (27.6)	
Senior high school	2277 (27.5)	1474 (28.6)	803 (25.7)	
College/university or higher	3776 (45.6)	2319 (45)	1457 (46.7)	
Occupation				0.006
Employed	2390 (28.9)	1455 (28.2)	935 (29.9)	
Own business	3044 (36.8)	1863 (36.1)	1181 (37.8)	
Others	2843 (34.3)	1837 (35.6)	1006 (32.2)	
Ability to pay for medication				<0.001
Very or fairly difficult	4475 (54)	2460 (47.7)	2015 (64.5)	
Very or fairly easy	3805 (46)	2698 (52.3)	1107 (35.5)	
Social status				<0.001
Low	1403 (16.9)	811 (15.7)	592 (19)	
Middle or high	6879 (83.1)	4349 (84.3)	2530 (81)	
BMI, kg/m^2^				0.063
Underweight (BMI < 18.5)	783 (9.5)	494 (9.6)	289 (9.3)	
Normal weight (18.5 ≤ BMI < 25.0)	6518 (78.8)	4020 (78)	2498 (80)	
Overweight/obese (BMI ≥ 25.0)	974 (11.8)	638 (12.4)	336 (10.8)	
Pre-existing health conditions				<0.001
Non-PEHC	6415 (77.4)	4494 (87.1)	1921 (61.4)	
PEHC	1876 (22.6)	668 (12.9)	1208 (38.6)	
Preventive behaviors				
Mask wearing				<0.001
Not adhering	2427 (55.8)	1174 (45.2)	1253 (71.5)	
Adhering	1921 (44.2)	1421 (54.8)	500 (28.5)	
Hand washing				<0.001
Not adhering	3291 (75.7)	1779 (68.6)	1512 (86.3)	
Adhering	1057 (24.3)	816 (31.4)	241 (13.7)	
Physical distancing				<0.001
Not adhering	3453 (79.4)	1893 (72.9)	1560 (89)	
Adhering	895 (20.6)	702 (27.1)	193 (11.0)	
HL index, mean ± SD	28.1 ± 9.4	30.1 ± 9.2	24.9 ± 8.9	<0.001

Abbreviations: S-COVID-19-S, suspected coronavirus disease 2019 symptoms; BMI, body mass index; PEHC, pre-existing health condition; HL, health literacy. * Results of Chi-square test, or analysis of variance (ANOVA) test, appropriately. ^†^ Suspected COVID-19 symptoms included fever, cough, dyspnea, myalgia, fatigue, sputum production, confusion, headache, sore throat, rhinorrhea, chest pain, hemoptysis, diarrhea, and nausea/vomiting.

**Table 2:** Associations of pre-existing health conditions, preventive behaviors, and health literacy with having S-COVID-19-S (*N* = 8291).

Variables	S-COVID-19-S *			
	Unadjusted OR (95% CI)	*p*	Adjusted OR (95% CI) ^†^	*p*
Pre-existing health condition				
Non-PEHC	1.00		1.00	
PEHC	4.23 (3.80, 4.72)	<0.001	3.38 (3.01, 3.79)	<0.001
Preventive behaviors				
Mask wearing				
Not adhering	1.00		1.00	
Adhering	0.33 (0.29, 0.38)	<0.001	0.36 (0.31, 0.41)	<0.001
Hand washing				
Not adhering	1.00		1.00	
Adhering	0.35 (0.30, 0.41)	<0.001	0.38 (0.32, 0.45)	<0.001
Physical distancing				
Not adhering	1.00		1.00	
Adhering	0.33 (0.28, 0.40)	<0.001	0.35 (0.29, 0.42)	<0.001
HL index, 1-score increment	0.94 (0.93, 0.94)	<0.001	0.95 (0.94, 0.96)	<0.001

Abbreviations: OR, odds ratio; CI, confidence interval; S-COVID-19-S, suspected coronavirus disease 2019 symptoms; PEHC, pre-existing health condition; HL, health literacy. * Suspected COVID-19 symptoms including fever, cough, dyspnea, myalgia, fatigue, sputum production, confusion, headache, sore throat, rhinorrhea, chest pain, hemoptysis, diarrhea, and nausea/vomiting. ^†^ Adjusted for age, occupation, ability to pay for medication, social status, and body mass index.

**Table 3:** Interactions of health literacy and preventive behaviors with pre-existing health conditions on suspected COVID-19 symptoms (*N* = 8291).

Interaction	S-COVID-19-S ^†^			
	Model 1		Model 2	
	OR (95% CI)	*p*	OR (95% CI)	*p*
PEHC and HL				
Non-PEHC × Lowest HL	1.00		1.00	
PEHC × Lowest HL	14.16 (9.85, 20.35)	<0.001	16.53 (11.41, 23.96)	<0.001
Non-PEHC × HL with 1-score increment	0.97 (0.96, 0.97)	<0.001	0.98 (0.97, 0.98)	<0.001
PEHC × HL with 1-score increment	0.94 (0.93, 0.96)	<0.001	0.93 (0.92, 0.94)	<0.001
PEHC and mask wearing				
Non-PEHC × Not adhering	1.00		1.00	
PEHC × Not adhering	19.57 (15.54, 24.64)	<0.001	17.24 (13.58, 21.88)	<0.001
Non-PEHC × Adhering	0.67 (0.57, 0.79)	<0.001	0.65 (0.55, 0.77)	<0.001
PEHC × Adhering	0.20 (0.14, 0.29)	<0.001	0.23 (0.16, 0.32)	<0.001
PEHC and hand washing				
Non-PEHC × Not adhering	1.00		1.00	
PEHC × Not adhering	11.67 (9.76, 13.97)	<0.001	10.31 (8.53, 12.46)	<0.001
Non-PEHC × Adhering	0.51 (0.42, 0.63)	<0.001	0.50 (0.40, 0.61)	<0.001
PEHC × Adhering	0.64 (0.43, 0.97)	0.035	0.78 (0.51, 1.19)	0.250
PEHC and physical distancing				
Non-PEHC × Not adhering	1.00		1.00	
PEHC × Not adhering	11.99 (10.03, 14.34)	<0.001	10.54 (8.73, 12.73)	<0.001
Non-PEHC × Adhering	0.43 (0.34, 0.54)	<0.001	0.41 (0.32, 0.51)	<0.001
PEHC × Adhering	0.67 (0.44, 1.02)	0.063	0.82 (0.53, 1.27)	0.376

Abbreviations: OR, odds ratio; CI, confidence interval; S-COVID-19-S, suspected coronavirus disease 2019 symptoms; PEHC, pre-existing health conditions; HL, health literacy. Model 1: Interaction between PEHC and health literacy, preventive behaviors. Model 2: Adjusted for age, occupation, ability to pay for medication, social status, and body mass index. ^†^ Suspected COVID-19 symptoms including fever, cough, dyspnea, myalgia, fatigue, sputum production, confusion, headache, sore throat, rhinorrhea, chest pain, hemoptysis, diarrhea, and nausea/vomiting.

## Supplementary Materials

The following are available online at https://www.mdpi.com/1660-4601/17/22/8598/s1, Table S1. Association between socio-demographic characteristics and having S-Covid-19-S in studied participants via logistic regression (*N* = 8291). Table S2. Spearman correlation among covariates.
