# Supplementary material for: Health Literacy and Preventive Behaviors Modify the Association between Pre-Existing Health Conditions and Suspected COVID-19 Symptoms: A Multi-Institutional Survey
Source: Int J Environ Res Public Health. 2020 Nov 19;17(22):8598. doi: 10.3390/ijerph17228598 (PMC7699410; doi:10.3390/ijerph17228598)
Supplement: Supplementary file 1 [file ijerph-17-08598-s001.pdf]

**Table 1.** Association between Socio-demographic Characteristics and Having S-Covid-19-S in Studied Participants via Logistic Regression (*N* = 8291).

| Variables | Have S-Covid-19-S † | |
|---|---|---|
| | OR (95% CI) | *p* |
| Age, year | | |
| 18–39 | 1.00 | |
| 40–59 | 1.67 (1.50, 1.85) | <0.001 |
| 60–85 | 3.03 (2.70, 3.40) | <0.001 |
| Gender | | |
| Women | 1.00 | |
| Men | 1.01 (0.92, 1.10) | 0.837 |
| Marital status | | |
| Never married | 1.00 | |
| Ever married | 2.05 (1.81, 2.31) | <0.001 |
| Education attainment | | |
| Junior high school or below | 1.00 | |
| Senior high school | 0.86 (0.76, 0.97) | 0.015 |
| College/university or higher | 0.99 (0.89, 1.10) | 0.884 |
| Occupation | | |
| Employed | 1.00 | |
| Own business | 0.99 (0.88, 1.10) | 0.808 |
| Others | 0.85 (0.76, 0.95) | 0.005 |
| Ability to pay for medication | | |
| Very or fairly difficult | 1.00 | |
| Very or fairly easy | 0.50 (0.46, 0.55) | <0.001 |
| Social status | | |
| Low | 1.00 | |
| Middle or high | 0.80 (0.71, 0.90) | <0.001 |
| BMI, kg/m$^2$ | | |
| Underweight (BMI < 18.5) | 0.94 (0.81, 1.10) | 0.441 |
| Normal weight (18.5 ≤ BMI < 25.0) | 1.00 | |
| Overweight/obese (BMI ≥ 25.0) | 0.85 (0.74, 0.98) | 0.022 |

Abbreviations: OR, odds ratio; CI, confidence interval; S-COVID-19-S, suspected corona virus disease-2019 symptoms; BMI, body mass index. † Suspected COVID-19 symptoms including fever, cough, dyspnea, myalgia, fatigue, sputum production, confusion, headache, sore throat, rhinorrhea, chest pain, hemoptysis, diarrhea, and nausea/vomiting.

**Table 2.** Spearman Correlation among Covariates (*N*=8291).

| | Age | Gender | Marital status | Education | Occupation | Ability to pay | Social status |
|---|---|---|---|---|---|---|---|
| Gender | 0.04 | | | | | | |
| Marital status | 0.38 | −0.06 | | | | | |
| Education | −0.41 | 0.04 | −0.27 | | | | |
| Occupation | 0.10 | −0.07 | −0.16 | −0.26 | | | |
| Ability to pay | −0.17 | −0.01 | −0.08 | 0.19 | −0.17 | | |
| Social status | −0.10 | 0.02 | −0.01 | 0.21 | −0.14 | 0.29 | |
| Body mass index | 0.05 | 0.06 | 0.13 | −0.01 | -0.08 | 0.03 | 0.04 |
